# Long‐Term Care of OLP Patients: A Retrospective Study From a Single Hospital Outpatient Clinic

**DOI:** 10.1111/jop.70179

**Published:** 2026-09-04

**Authors:** F. Scilla, G. Gioco, L. Ferrero, R. Castagnola, A. Zafarone, L. Cimino, M. Tranfa, C. Rupe, C. Lajolo

**Affiliations:** ^1^ Head and Neck Department Fondazione Policlinico Universitario A. Gemelli‐IRCCS, School of Dentistry, Università Cattolica del Sacro Cuore Rome Italy

## Abstract

**Background:**

Oral lichen planus (OLP) is a chronic immune‐mediated disorder with malignant potential. However, its real‐world clinical course remains incompletely characterized. This study aimed to describe the natural clinical–histopathological trajectory of OLP in a single tertiary centre and to identify factors associated with dysplasia and malignant transformation over time.

**Methods:**

We conducted a single‐centre retrospective cohort study including patients diagnosed with OLP and followed between 2007 and 2024 at the Oral Medicine Unit of Fondazione Policlinico Universitario A. Gemelli IRCCS. Demographic, clinical (red vs. white phenotype and lesion distribution), microbiological (Candida spp.), therapeutic, and histopathological variables were collected across follow‐up. Univariate and multivariable logistic regression models were used to evaluate factors associated with dysplasia and malignant transformation.

**Results:**

Among 308 screened patients, 246 met inclusion criteria (173 women, 70.3%; mean age 63.2 ± 15.0 years) with a mean follow‐up of 55.5 ± 37.0 months. At baseline, Candida spp. colonization was detected in 55 patients (22.4%) and was more frequent in red‐type OLP than white‐type lesions (33.3% vs. 17.0%; OR 2.44, 95% CI 1.32–4.53; *p* = 0.005). During follow‐up, 33 patients (13.4%) developed fungal infection despite an initially negative swab. Epithelial dysplasia emerged in 42 patients (17.1%). In multivariable analysis, dysplasia was independently associated with increasing age (OR 1.06, 95% CI 1.03–1.10; *p* = 0.0001) and red‐type OLP(OR 2.24, 95% CI 1.10–4.55; *p* = 0.026). Eight patients (3.25%) developed infiltrative OSCC after a mean interval of 28.3 months from OLP diagnosis (range 4–55).

**Conclusions:**

In this long‐term single‐centre cohort, OLP frequently evolved through dysplasia during follow‐up, and malignant transformation occurred in a minority of patients. Older age and red‐type phenotype characterized patients more likely to develop dysplasia, while malignant transformation was more frequent in the case of previously documented dysplastic lesions, in keeping with the notion of dysplasia as a step along the carcinogenic pathway, older age, and diffuse lesions.

## 1 Introduction

Oral Lichen Planus (OLP) is a chronic inflammatory mucocutaneous disease of unknown etiology, primarily affecting the oral mucosa [1]. Global prevalence ranges from 1.01% to 3% [2, 3, 4], with an estimated 1.43% prevalence in Europe [5]. While its precise cause remains unclear, it is thought to result from a T‐cell mediated response, triggered by microbial agents, chemicals, stress, or viral infections [6].

Clinically, OLP typically presents bilaterally and symmetrically, with the buccal mucosa as the most frequently affected site [7]. The most characteristic clinical feature is the presence of hyperkeratotic white reticular patterns, sometimes accompanied by atrophic, erosive, plaque‐like, or bullous lesions [8].

The clinical course of OLP is characterized by alternating periods of exacerbation and remission [9]. During flare‐ups, symptoms such as pain and inflammation intensify, while during remission, symptoms typically subside, improving the patient's quality of life [10]. The management of OLP is complex due to its unpredictable course and the increased risk of malignancy emphasizing the importance of regular oral cancer screenings [11]. In fact, OLP is classified as an Oral Potentially Malignant Disorder (OPMD) [12], with an estimated risk of malignant transformation ranging from 0.4% to 5.98% [13, 14]. These data reveal how the OLP transformation rate remains unclear, largely due to overly restrictive diagnostic criteria which conduct to an underrated number of diagnoses, short follow‐up durations, and the poor quality of some published studies [15]. Similarly, several risk factors have been advocated to be involved in OLP malignant transformation, including the erosive phenotype, tongue involvement, female sex, and advanced age [16]. Nevertheless, no robust evidence has clarified the impact of the different risk factors on OLP malignant transformation. Furthermore, over the years, multiple controversies have surrounded the interpretation of oral epithelial dysplasia, which many authors have regarded as an exclusion criterion for the diagnosis of OLP [17]. The long‐standing consideration of epithelial dysplasia as an exclusion criterion for OLP diagnosis has led to the misclassification of clinically significant cases, ultimately contributing to a marked underestimation of the malignant transformation potential of this disorder. In contrast, a recent position paper has emphasized the importance of including epithelial dysplasia in the assessment of OLP, recognizing it as a key histopathological feature that may reflect disease progression and better predict malignant potential [18].

The primary aim of this paper was to evaluate the rate of malignant transformation among a cohort of patients affected by oral lichen planus with a minimum 6‐month follow‐up. Secondary aims were: (a) To study and describe the characteristics of these patients to identify potential risk factors for oral dysplasia and malignant transformation, (b) to evaluate the therapeutic effects and features of OLP drugs, (c) to provide a comprehensive characterization of the demographic and clinical profile of a well‐defined cohort of patients diagnosed with oral lichen planus.

## 2 Materials and Methods

### 2.1 Study Design and Setting

This retrospective observational study analyzed a population of patients affected by OLP, enrolled in a regular follow‐up program at the Oral Medicine Department of Fondazione Policlinico Universitario Agostino Gemelli between January 2007 and October 2024.

The study was approved by the Ethics Committee of the Università Cattolica del Sacro Cuore (ID‐7603) and registered at ClinicalTrials.gov (ID: NCT06934863).

This study was conducted according to the Declaration of Helsinki and carried out according to the Strobe guidelines. All patients signed an informed consent.

### 2.2 Outcome

The main outcome of this study was to evaluate the rate of malignant transformation among a cohort of patients with a clinical‐pathological OLP diagnosis, with a minimum follow‐up of 6 months.

Secondary outcomes were to describe the long‐term clinical and pathological course of the disease, evaluating the risk factors for malignant transformation, as well as the impact of oral candidiasis on OLP behaviour, and. to evaluate the effects of different OLP treatments.

### 2.3 Patients Selection

Inclusion Criteria—Clinical and histological diagnoses of OLP made according to the WHO diagnostic criteria modified by van der Meij and van der Waal [19].

- Minimum 6 months follow‐up.

#### 2.3.1 Exclusion Criteria

- Patients with oral lichenoid lesions (OLL) were excluded, according to the recent WHO classification of OPMD (Warnakulasuriya et al., 2021) [20].
- Patients with anamnesis of hematopoietic stem cell transplant
- Patients with an OSCC at the first histological examination concomitant with OLP diagnosis.

The clinical diagnosis of OLP was achieved using the following criterion: multifocal, symmetric distribution; white and red lesions exhibiting one or more of the reticular/papular, atrophic, erosive, plaque, bullous forms. The histopathological criteria were as follows: (1) A well‐defined band‐like zone of cellular infiltration confined to the superficial part of the connective tissue, consisting mainly of lymphocytes; (2) signs of liquefaction degeneration in the basal cell layer; and (3) absence of epithelial dysplasia. A final diagnosis of OLP was made, fulfilling both the clinical and histopathological criteria.

### 2.4 Variables

Demographic and clinical data from recent and remote anamnesis including sex, age, systemic diseases, pharmacological anamnesis, smoking habits, and related medications were collected.

Smokers were categorized as light (< 15 cigarettes/day), heavy (> 15 cigarettes/day), and former smokers, defined as those who quit smoking less than 5 years before the initial visit [21]. Moreover, smoking was considered a dichotomous variable, grouping former, light, and heavy smokers.

Comorbidities with reported association to oral lichen planus were also collected (i.e., hyperthension [22], Hashimoto thyroidits [23], psoriasis [21], diabetes [24], hyperthyroidism [25]).

The clinical forms of OLP were classified into the following types: (i) Reticular, characterized by the presence of nonremovable, lacelike keratotic lesions on the oral mucosa; (ii) plaque‐like, with a dense thickening of the mucosal tissue; (iii) erosive, with well‐defined erosions; (iv) atrophic, with the regions of thinned/atrophic epithelium within previously defined keratotic lesions; (v) bullous type, with blister formation. Moreover, the clinical forms were also collected in a dichotomous form: the atrophic‐erosive forms were classified as red forms; the reticular and plaque forms as white forms [26, 27].

Data about oral and extraoral involvement were also collected. OLP was classified as localized when lesions were confined to a single anatomical site (e.g., tongue, gingiva, or buccal mucosa), and as diffuse when multiple oral sites were simultaneously affected.

Symptomatology related to oral lichen planus was recorded and categorized as follows: symptomatic at diagnosis, asymptomatic at diagnosis, asymptomatic throughout follow‐up, or symptoms developed during follow‐up.

Data regarding the clinical evolution of OLP, the occurrence of dysplasia, as well as the malignant transformation of lesions into oral squamous cell carcinoma (OSCC), were also recorded. The epithelial dysplasia was classified according to the WHO revised grading system for oral epithelial dysplasia (2017) [28]. The OLP cohort which developed OSCC from OLP will be mentioned as t‐OLP (transformed‐OLP) throughout the paper.

Candida spp. infection and antimycotic therapy were also retrieved through the medical records. In particular, oral colonization by *Candida* spp. was recorded using a sterile swab (eSwab, Copan's Liquid Amies Elution Swab, Copan Italia SPA, Brescia, Italy); it was rubbed on the mucosal surfaces of the oral cavity presenting oral lesions. Post‐sample collection, sterile swabs were placed in tubes containing 1 mL of transport medium. The tubes were then stored at 4°C until further processing, which was always performed within 12 h from the oral swab collection. Processing involved streak inoculation of the swab onto Sabouraud dextrose agar (SDA) plates, followed by incubation at 37°C for 48 h, according to the manufacturer's instructions [29].

Specifically, the Candida spp. infection was investigated in three different moments in the management of OLP disease according to Jainkittivong et al. [30]: (i) At the first visit with a suspected diagnosis of OLP; (ii) every 6 months during the follow‐up of the disease; (iii) before the immunosuppressive therapy. For the purpose of this study, the following definitions were applied: colonization indicated an isolated positive Candida spp. swab in the absence of clinical signs of infection; candidiasis (or Candida infection) indicated a positive swab accompanied by clinically consistent mucosal findings; and re‐colonization indicated a new positive swab obtained during follow‐up in a patient with a previously negative baseline swab.

In case of positive swab, the first‐line treatment consisted of topical nystatin. The second‐line treatment involved the administration of systemic fluconazole [31]. Antifungal therapy was administered to both symptomatic individuals and asymptomatic subjects exhibiting clinical evidence of oral candidiasis [32].

The therapeutic management of oral lichen planus primarily consists of first‐line treatment with topical corticosteroids, whereas systemic corticosteroids or other immunosuppressive agents are considered as second‐line options [33]. Clobetasol propionate ointment (5 mg, approximately corresponding to the amount contained in a coffee spoon) was applied directly to the affected areas of the oral mucosa and retained in the mouth for 5 min before expelling. The treatment was administered three times daily, then progressively tapered to once every other day, gradually tapered until the minimum effective dose required to control symptoms was reached [34].

Patients were defined as “Responder” in case of symptoms resolution or complete remission. All clinical evaluations throughout the entire study period were performed by a single senior clinician with specific expertise in oral medicine, thus minimising inter‐observer variability in the interpretation of both symptoms and clinical signs. At each visit, treatment response was assessed through a standardised structured evaluation combining the patient‐reported symptom status and the objective clinical appearance of the lesions (extent and type of lesion and change relative to baseline), supported by photographic documentation allowing direct comparison over time; accordingly, a patient was classified as a “Responder” only in case of concordant improvement of both the subjective and the objective components.

Treatment was restarted upon recurrence of symptoms or lesions. In non‐responders, various alternative treatment strategies were employed, including systemic corticosteroids, topical ciclosporin, tacrolimus, or their combination.

Furthermore, during treatment with topical corticosteroids, patients underwent a one‐week course of topical antifungal therapy to reduce the risk of superinfection with Candida spp. [35].

Other clinical data (i.e., frequency and site of biopsy) were also collected.

### 2.5 Sample Size and Statistical Analysis

The sample size was calculated using the sample size formula for one‐proportion test. Assuming a potential OLP transformation rate at 3%, aiming for a precision of 5% and with a power of 80,236 patients were needed to be included in the final sample.

Qualitative variables were described using absolute and percentage frequencies, whereas quantitative variables were summarized as mean and standard deviation (SD). The Kolmogorov–Smirnov test was performed to evaluate the normal distribution of quantitative variables.

Correlation analyses were performed to evaluate the differences between red and white forms of OLP.

Mann–Whitney U test and Kruskal–Wallis test were performed to compare continuous variables with nonparametric distributions between red and white forms of OLP. Parametric variables were analyzed using ANOVA. Pearson's χ2 test and Fisher's exact test were utilized to compare discontinuous variables between groups.

As a secondary, explorative analysis, univariate analyses were performed to identify risk factors associated with the following outcomes: malignant transformation (primary outcome), epithelial dysplasia, *Candida* spp. re‐colonization, incomplete response to treatment (non‐responder). These risk factors were then introduced into stepwise logistic regression analyses to identify independent predictors of the outcomes. These analyses were only performed for outcomes with an adequate number of events (10 events‐per‐variable threshold). The level of significance (*p* > 0.05) was considered as exclusion criterion for the stepwise backward analysis.

The incidence rate of malignant transformation was calculated as the number of events divided by the total person‐time at risk and expressed per 100 person‐years.

Kaplan–Meier analysis for malignant transformation was performed according to variables significantly associated with the main outcome, and survival distributions were compared using the log‐rank (Mantel‐Cox) test.

All statistical analyses were performed using the IBM SPSS Statistics software (IBM Corp. Released 2017. IBM SPSS Statistics for Apple, Version 25.0; Armonk, NY: IBM Corp).

## 3 Results

### 3.1 Participants Enrollment

Among the 308 suspicious OLP patients assessed for eligibility, a total of 62 patients were excluded because they did not meet the inclusion criteria, as specified in the STROBE flow‐chart (Figure 1).

**FIGURE 1:**
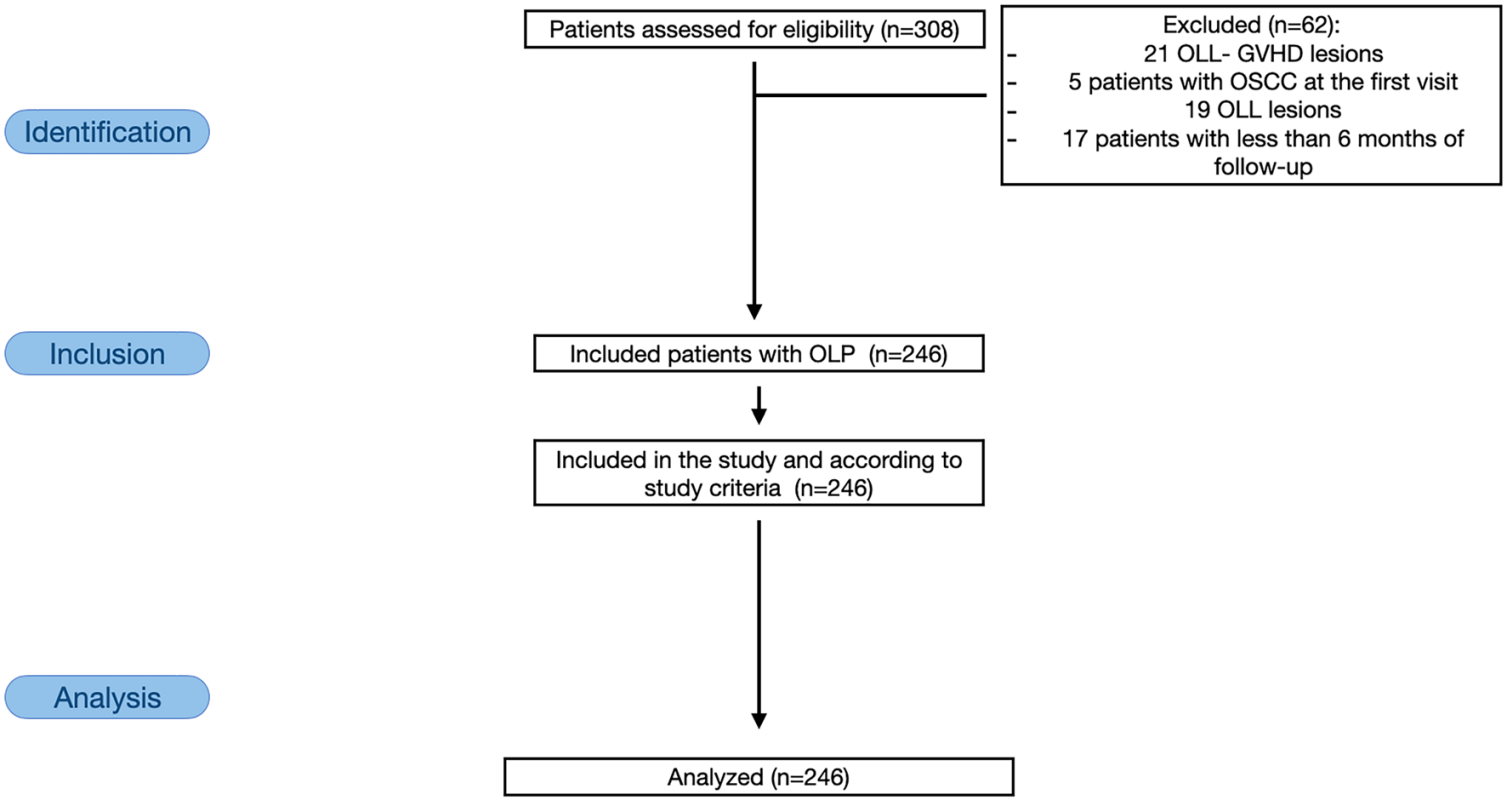
STROBE flow chart.

Thus, 246 patients were included, with a female–male ratio of 2.37 (173 female—70.33% and 73 male—29.67%) with a mean follow‐up of 55.48 months (SD: 37.0). The cohort's mean age was 63.2 years (SD: 14.98). Clinical characteristics of the sample are presented in Table 1, while Table 2 reports the demographic characteristics of the sample, stratified for OLP type (red or white).

**TABLE 1:** Clinical characteristics of the included sample.

Variable		Total
Sex	Men	73 (29.7%)
	Women	173 (70.3%)
	Total	246 (100%)
Age at diagnosis (years)	Mean (Range, SD)	63.2 (13–95; SD: 14.98)
Age at diagnosis (decades)	0–49	40 (16.3%)
	50–59	43 (17.5%)
	60–69	63 (25.6%)
	70–79	69 (28.1%)
	Over 80	31 (12.6%)
	Total	246 (100%)
OLP Type	Reticular	72 (29.3%)
	Plaque	93 (37.8%)
	Erosive	53 (21.6%)
	Atrophic	26 (10.6%)
	Bullous	2 (0.8%)
	Total	246 (100%)
OLP Type (Dichotomous)	White	165 (67.1%)
	Red	81 (32.9%)
	Total	246 (100%)
OLP lesions	Localized	86 (34.9%)
	Diffuse	160 (65.1%)
	Total	246 (100%)
OLP localization^b^	Gingiva	145 (58.9%)
	Buccal mucosa	155 (62.9%)
	Tongue	138 (56.1%)
	Palate	84 (34.2%)
	Mouth floor	69 (28.1%)
	Lips	36 (14.6%)
Other districts	Cutaneous	43 (17.5%)
	Genital	12 (4.9%)
	Both	11 (4.5%)
	No	180 (73.1%)
	Total	246 (100%)
Smoking	Former smokers	26 (10.6%)
	Light smokers	25 (10.2%)
	Heavy Smokers	21 (8.5%)
	Non smokers	174 (70.7%)
	Total	246 (100%)
Comorbidities	None	95 (38.6%)
	One	64 (26%)
	Two or more	87 (35.4%)
	Total	246 (100%)
Candida spp. swab (baseline)	Negative	191 (77.6%)
	Positive	55 (22.4%)
	Total	246 (100%)
Candida spp. swab (re‐colonization)	Negative	213 (86.6%)
	Positive	33 (13.4%)
	Total	246 (100%)
Candida therapy	No	179 (72.8%)
	Fluconazole	19 (7.7%)
	Nystatin	41 (16.7%)
	Both	7 (2.8%)
	Total	246 (100%)
Symptoms	Yes	146 (59.4%)
	No	100 (40.6%)
	Total	246 (100%)
Symptoms	Mild	137 (93.8%)
	Severe	9 (6.2%)
	Total^a^	146 (100%)
Symptoms	At diagnosis	92 (63.1%)
	During follow‐up	54 (36.9%)
	Total^a^	146 (100%)
OLP Treatment	Yes	118 (47.9%)
	No	128 (52.1%)
	Total	246 (100%)
Responder status	Yes	73 (61.9%)
	No	45 (38.1%)
	Total^c^	118 (100%)
Follow‐up (months)	Mean (Range, SD)	55.48 (6–210; SD: 37.00)
Number of biopsies	Mean (Range, SD)	1.94 (1–8; SD: 1.24)
Number of biopsies	Less than 2	181 (73.6%)
	More than 2	65 (26.4%)
	Total	246 (100%)
Presence of dysplastic lesions	Yes	42 (17.1%)
	No	204 (82.9%)
	Total	246 (100%)
Malignant Transformation	Yes	8 (3.3%)
	No	238 (96.7%)
	Total	246 (100%)

*Note: Mild symptoms*: Burning sensations, when consuming spicy or acidic foods, metallic taste, mucosal roughness or discomfort during oral hygiene practices; *Severe symptoms:* feeding difficulties that required dietary modifications and/or analgesic therapy.

^a^ Total number of patients who reported symptoms throughout the course of the study.

^b^ Percentages are calculated on the whole sample, the total exceeds 100% considering that each patient may have more than one oral site affected by OLP.

^c^ Total number of patients receiving OLP treatment.

**TABLE 2:** Characteristics of the included sample, stratified for red and white forms of OLP and results of the statistical analysis for differences between groups.

Variable		White OLP	Red OLP	Total	Statistical significance
Sex	Men	51 (30.9%)	22 (27.2%)	73 (29.7%)	
	Women	114 (69.1%)	59 (72.8%)	173 (70.3%)	χ2 Test—*p* = 0.656
	Total	165 (100%)	81 (100%)	246 (100%)	
Age (years)	Mean (Range, SD)	61.64 (13–88; SD: 15.11)	66.53 (28–95; SD: 14.25)	63.2 (13–95; SD: 14.98)	**Mann Whitney—*p* = 0.01**
Age at diagnosis (decades)	0–49	30 (18.2%)	10 (12.3%)	40 (16.3%)	χ2 Test—*p* = 0.222
	50–59	29 (17.6%)	14 (17.3%)	43 (17.5%)	
	60–69	46 (27.9%)	17 (20.9%)	63 (25.6%)	
	70–79	44 (26.7%)	25 (30.9%)	69 (28.1%)	
	Over 80	16 (9.7%)	15 (18.5%)	31 (12.6%)	
	Total	165 (100%)	81 (100%)	246 (100%)	
OLP lesions	Localized	60 (36.4%)	26 (32.1%)	86 (34.9%)	χ2 Test—*p* = 0.570
	Diffuse	105 (63.6%)	55 (68.9%)	160 (65.1%)	
	Total	165 (100%)	81 (100%)	246 (100%)	
OLP localization^a^	Gingiva	94 (56.9%)	51 (62.9%)	145 (58.9%)	χ2 Test—*p* = 0.409
	Buccal mucosa	103 (62.4%)	52 (64.2%)	155 (50.8%)	χ2 Test—*p* = 0.892
	Tongue	92 (55.8%)	46 (56.8%)	138 (56.1%)	χ2 Test—*p* = 0.892
	Palate	44 (26.7%)	40 (49.4%)	84 (34.2%)	χ2 Test—*p* = 0.675
	Mouth floor	48 (29.1%)	21 (25.9%)	69 (28.1%)	χ2 Test—*p* = 0.660
	Lips	23 (13.9%)	13 (16.0%)	36 (14.6%)	χ2 Test—*p* = 0.665
Other districts	Cutaneous	25 (15.5%)	18 (22.2%)	43 (17.5%)	χ2 Test—*p* = 0.397
	Genital	10 (6.1%)	2 (2.5%)	12 (4.9%)	
	Both	8 (4.8%)	3 (3.7%)	11 (4.5%)	
	No	122 (73.9%)	58 (71.6%)	180 (73.17%)	
	Total	165 (100%)	81 (100%)	246 (100%)	
Smoking	Former smokers	20 (12.1%)	6 (7.4%)	26 (10.57%)	χ2 Test—*p* = 0.439
	Light smokers	16 (9.7%)	9 (11.1%)	25 (10.16%)	
	Heavy Smokers	17 (10.3%)	4 (4.9%)	21 (8.54%)	
	Non‐Smokers	112 (67.9%)	62 (76.5%)	174 (70.73%)	
	Total	165 (100%)	81 (100%)	246 (100%)	
Comorbidities	None	69 (41.8%)	26 (32.1%)	95 (38.6%)	χ2 Test—*p* = 0.330
	One	44 (26.7%)	20 (24.7%)	64 (26%)	
	Two or more	52 (31.5%)	35 (43.2%)	87 (35.4%)	
	Total	165 (100%)	81 (100%)	246 (100%)	
Candida spp. swab	Negative	137 (83%)	54 (66.7%)	191 (77.6%)	**χ2 Test—*p* = 0.005**
	Positive	28 (17%)	27 (33.3%)	55 (22.4%)	
	Total	165 (100%)	81 (100%)	246 (100%)	
Candida spp. swab (re‐colonization)	Negative	152 (92.1%)	61 (75.3%)	213 (86.6%)	**χ2 Test—*p* = 0.001**
	Positive	13 (7.9%)	20 (24.7%)	33 (13.4%)	
	Total	165 (100%)	81 (100%)	246 (100%)	
Candida therapy	No	128 (77.6%)	51 (62.9%)	179 (72.8%)	χ2 Test—*p* = 0.08
	Fluconazole	11 (6.7%)	8 (9.9%)	19 (7.7%)	
	Nystatin	22 (13.3%)	19 (23.5%)	41 (16.7%)	
	Both	4 (2.4%)	3 (3.7%)	7 (2.8%)	
	Total	165 (100%)	81 (100%)	246 (100%)	
Symptoms	Yes	79 (47.9%)	67 (82.7%)	146 (59.4%)	
	No	86 (52.1%)	14 (17.3%)	100 (40.6%)	
	Total	165 (100%)	81 (100%)	246 (100%)	
OLP Treatment	Yes	66 (40%)	52 (64.2%)	118 (47.9%)	**χ2 Test—*p* = 0.001**
	No	99 (60%)	29 (35.8%)	128 (52.1%)	
	Total	165 (100%)	81 (100%)	246 (100%)	
Responder status	Yes	40 (61.5%)	33 (62.3%)	73 (61.9%)	χ2 Test—*p* = 0.935
	No	25 (38.5%)	20 (37.6%)	45 (38.1%)	
	Total	65 (100%)	53 (100%)	118 (100%)^b^	
Number of biopsies	Mean (Range, SD)	1.87 (1–6; SD:1.14)	2.11 (1–8; SD: 1.4)	1.94 (1–8; SD: 1.24)	Mann Whitney—*p* = 0.147
Number of biopsies	Less than 2	125 (75.8)	56 (69.1%)	181 (73.6%)	χ2 Test—*p* = 0.284
	More than 2	40 (24.2%)	25 (30.9%)	65 (26.4%)	
	Total	165 (100%)	81 (100%)	246 (100%)	
Follow‐up (months)	Mean (Range, SD)	52.58 (6–210; SD: 36.00)	61.35 (6–183; SD: 38.52)	55.48 (6–210; SD: 37.00)	Mann Whitney—*p* = 0.002
Presence of dysplastic lesions	Yes	20 (12.1%)	22 (27.2%)	42 (17.1%)	**χ2 Test—*p* = 0.006**
	No	145 (87.9%)	59 (72.8%)	204 (82.9%)	
	Total	165 (100%)	81 (100%)	246 (100%)	
Malignant Transformation	Yes	4 (2.4%)	4 (4.9%)	8 (3.3%)	**χ2 Test—*p* = 0.008**
	No	161 (97.6%)	77 (95.1%)	238 (96.7%)	
	Total	165 (100%)	81 (100%)	246 (100%)	

^a^ Percentages are calculated on the whole sample, the total exceeds 100% considering that each patient may have more than one oral site affected by OLP.

^b^ Total number of patients receiving OLP treatment.

At baseline, *Candida* spp. was positive in 55 patients (22.4%). Among the 55 patients with baseline Candida spp. positivity, approximately 11 reported recent systemic corticosteroids use prior to clinical assessment, and 6 were wearing complete dentures. No other evident risk factors for oral candidiasis were identified in the remaining patients. Patients suffering from red forms of OLP showed a higher rate of *Candida* spp. colonization (33.3%) when compared to white forms (17%, OR: 2.44, 95% CI: 1.32–4.53, χ2 Test—*p* = 0.005). All cases of candidiasis were treated with antifungal therapy. Only two cases proved refractory to all treatments, including both topical (mouth rinse) and systemic antifungal regimens, with persistently positive *Candida Albicans* cultures during follow‐up. During the follow‐up, 33 patients (13.41%) developed a fungal infection, despite an initial negative swab and topical antifungal therapy to reduce the risk of superinfection.

The following risk factors for *candida* spp. re‐colonization were retrieved at multiple logistic regression: age (OR: 1.05, 95% CI: 1.01–1.09, *p* = 0.005), immunosuppressive treatment (OR: 8.3, 95% CI: 2.71–25.58, *p* = 0.0001), red forms of OLP (OR compared to white forms: 2.34, 95% CI: 1.02–5.33, *p* = 0.043). However, all these cases showed resolution after antimycotic therapy.

In the cohort, 118 patients (47.9% of the population) were treated with topical corticosteroids during their follow‐up. Ten patients developed limited adverse effects: mild gastrointestinal symptoms (5 patients), slight female hirsutism (2 patients), moon facies (2 patients), and oral burning sensations (1 patient). A total of 45 patients were defined as non‐responders to OLP treatment (38.14% of the ones receiving immunosuppressive treatments).

Red forms of OLP required immunosuppressive therapy more often than white forms (64.2% versus 40%, OR: 2.64, 95% CI: 1.52–4.58, χ2 Test—*p* = 0.001). However, there were no differences in terms of response to immunosuppressive treatment.

Logistic regression showed that non‐responders were frequently affected by multiple localizations of OLP lesions (OR versus single localizations: 3.04, 95% CI: 1.43–6.46, *p* = 0.004) and were positive to *Candida* spp. at baseline (OR: 2.34, 95% CI: 1.18–4.63, *p* = 0.014).

Forty‐two patients (17.1%) showed histopathological evidence of epithelial dysplasia during the follow‐up. The mean follow‐up for these patients was 71.7 months. Results of the univariate analysis of risk factors for dysplasia are reported in Supporting Information. Notably, 13 patients (30.95%) exhibited progression of lesion severity over time, with histopathologic grading evolving from mild to moderate dysplasia in subsequent biopsies. None of the patients who were diagnosed with dysplasia exhibited regression of the histological grade over time. In all cases, the dysplasia either persisted or progressed toward more severe forms. Dysplastic changes involving multiple anatomical locations, such as gingiva, buccal mucosa, and dorsum of the tongue, were also observed in 4 cases.

The logistic regression analysis (Table 3) highlighted the following risk factors for epithelial dysplasia: age (OR: 1.06, 95% CI: 1.03–1.10, *p* = 0.0001) and type of OLP (OR for red forms: 2.24, 95% CI: 1.10–4.55, *p* = 0.026).

**TABLE 3:** Summary of key risk estimates stratified for dysplasia.

Predictors	OR	95% CI	*p*
Dysplasia			
**Age** (Years)	1.063	1.031–1.096	**0.0001**
**OLP type**			
*White*	1.0 (Ref)		
*Red*	2.238	1.100–4.554	**0.026**

During the follow‐up, 8 patients developed infiltrative OSCC; thus, the overall transformation rate in the present cohort was 3.25%. Supporting Information report demographic and clinical data of the 8 patients who developed OSCC (t‐OLP cohort). The mean follow‐up duration of the t‐OLP cohort was 57.25 months (26–104; SD: 27.83), without significant differences with the non‐transformed cohort (*p* = 0.86). The mean time‐lapse between the diagnosis of OLP and the malignant transformation was 28.25 months (range: 4–55, SD: 17.86). Instead, among the four patients with prior dysplasia, the mean interval between the first histological diagnosis of dysplasia and the onset of OSCC was 39 months (min.‐max. 13–68 months).

The results of the univariate analysis stratified for malignant transformation are displayed in Table 4. The following variables showed statistical significance throughout the analysis: older age (Mann Whitney—*p* = 0.037), presence of previous dysplasia in the site of transformation (χ2 Test—*p* = 0.03), diffuse OLP lesions (χ2 Test—*p* = 0.05), and the number of biopsies (Mann Whitney—*p* < 0.001).

**TABLE 4:** Univariate statistical analysis stratified for malignant transformation.

Variable		Non transformed	t‐OLP	Total	Statistical significance
Sex	Men	72 (30.3%)	1 (12.5%)	73 (29.7%)	
	Women	166 (69.7%)	7 (87.5%)	173 (70.3%)	χ2 Test—*p* = 0.442
	Total	238 (100%)	8 (100%)	246 (100%)	
Age (years)	Mean (Range, SD)	63.25 (13–95; SD: 14.99)	74.13 (56–88; SD: 11.48)	63.2 (13–95; SD: 14.98)	**Mann Whitney—*p* = 0.037**
Age at diagnosis (decades)	0–49	40 (16.8%)	0 (−)	40 (16.3%)	χ2 Test—*p* = 0.235
	50–59	42 (17.6%)	1 (12.5%)	43 (17.5%)	
	60–69	61 (25.6%)	2 (25%)	63 (25.6%)	
	70–79	67 (28.2%)	2 (25%)	69 (28.1%)	
	Over 80	28 (11.8%)	3 (37.5%)	31 (12.6%)	
	Total	238 (100%)	8 (100%)	246 (100%)	
Follow‐up (months)	Mean (Range, SD)	54.85 (6–210; SD: 37.53)	57.25 (26–104; SD: 27.83)	55.48 (6–210; SD: 37.00)	Mann Whitney—*p* = 0.858
Presence of dysplastic lesions	Yes	38 (15.9%)	4 (50%)	42 (17.1%)	**χ2 Test—*p* = 0.03**
	No	200 (84.1%)	4 (50%)	204 (82.9%)	
	Total	238 (100%)	8 (100%)	246 (100%)	
OLP Type	Reticular	72 (30.3%)	0 (−)	72 (29.3%)	χ2 Test—*p* = 0.450
	Plaque	89 (37.4%)	4 (50%)	93 (37.8%)	
	Erosive	52 (21.8%)	1 (12.5%)	53 (21.6%)	
	Atrophic	23 (9.7%)	3 (37.5%)	26 (10.6%)	
	Bullous	2 (0.8%)	0 (−)	2 (0.8%)	
	Total	238 (100%)	8 (100%)	246 (100%)	
OLP lesions	Localized	86 (36.1%)	0 (−)	86 (34.9%)	
	Diffuse	152 (63.9%)	8 (100%)	160 (65.1%)	**χ2 Test—*p* = 0.05**
	Total	238 (100%)	8 (100%)	246 (100%)	
OLP localization	Gingiva	139 (58.40%)	6 (75%)	145 (58.94%)	χ2 Test—*p* = 0.485
	Buccal mucosa	149 (62.61%)	6 (75%)	155 (62.99%)	χ2 Test—*p* = 0.056
	Tongue	133 (55.88%)	5 (62.5%)	138 (56.10%)	χ2 Test—*p* = 0.505
	Palate	81 (34.03%)	3 (37.5%)	84 (34.16%)	χ2 Test—*p* = 0.132
	Mouth floor	66 (27.73%)	3 (37.5%)	69 (28.05%)	χ2 Test—*p* = 0.914
	Lips	35 (14.71%)	1 (12.5%)	36 (14.63%)	χ2 Test—*p* = 0.111
Other districts	Cutaneous	43 (18.1%)	0 (−)	43 (17.5%)	χ2 Test—*p* = 0.482
	Genital	11 (4.6%)	1 (12.5%)	12 (4.9%)	
	Both	11 (4.6%)	0 (−)	11 (4.5%)	
Smoking	Former smokers	26 (10.9%)	0 (−)	26 (10.6%)	χ2 Test—*p* = 0.784
	Light smokers	25 (10.5%)	0 (−)	25 (10.2%)	
	Heavy Smokers	19 (7.9%)	2 (25%)	21 (8.5%)	
	Non smokers	168 (70.6%)	6 (75%)	174 (70.7%)	
	Total	238 (100%)	8 (100%)	246 (100%)	
Comorbidities	None	94 (39.5%)	1 (12.5%)	95 (38.6%)	χ2 Test—*p* = 0.767
	One	61 (25.6%)	3 (37.5%)	64 (26%)	
	Two or more	83 (34.9%)	4 (50%)	87 (35.4%)	
	Total	238 (100%)	8 (100%)	246 (100%)	
Candida spp. swab	Negative	186 (78.2%)	5 (62.5%)	191 (77.6%)	χ2 Test—*p* = 1.000
	Positive	52 (21.8%)	3 (37.5%)	55 (22.4%)	
	Total	238 (100%)	8 (100%)	246 (100%)	
Candida therapy	No	174 (73.1%)	5 (62.5%)	179 (72.8%)	χ2 Test—*p* = 0.668
	Fluconazole	18 (7.6%)	1 (12.5%)	19 (7.7%)	
	Nystatin	40 (16.8%)	1 (12.5%)	41 (16.7%)	
	Both	6 (2.5%)	1 (12.5%)	7 (2.8%)	
		238 (100%)	8 (100%)	246 (100%)	
Number of biopsies	Mean (Range, SD)	1.85 (1–8; SD: 1.27)	4.63 (2–9; SD: 2.18)	1.94 (1–8; SD: 1.24)	**Mann Whitney—*p* < 0.001**
Responder status	Yes	68 (61.3%)	5 (71.4%)	73 (61.9%)	χ2 Test—*p* = 0.746
	No	43 (38.7%)	2 (28.6%)	45 (38.1%)	
	Total	111 (100%)	7 (100%)	118 (100%)	

Logistic regression was not performed due to the limited number of events.

Two patients showed no dysplastic changes in any previous biopsies and progressed directly from OLP to OSCC. The remaining two patients had never undergone a biopsy of the area that later transformed malignantly. Figure 2 shows the results of the Kaplan Meier Analysis, stratified according to the initial presence of dysplasia. Patients with dysplasia showed a lower estimated transformation‐free survival; however, the difference was not statistically significant (log‐rank test: *p* = 0.204).

**FIGURE 2:**
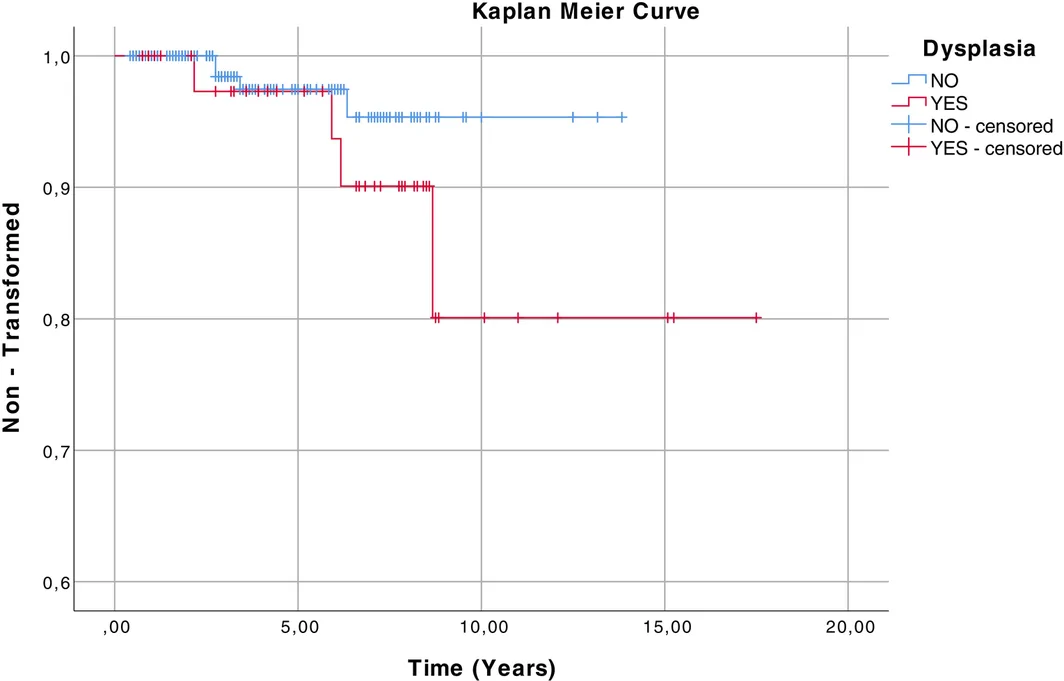
Kaplan Meier Analysis for malignant transformation, stratified according to the initial presence of dysplasia (log‐rank test: *p* = 0.204).

The incidence rate per 100 person‐years was 0.70.

Interestingly, three patients developed more than one primary OSCC in different sites: two patients were affected by three similar metachronous OSCCs involving the gingiva, floor of the mouth, and buccal mucosa (25%), while one patient developed a metachronous OSCC of the lateral border of the tongue and floor of the mouth (12.5%). However, no specific oral sites was a risk factor for the development of OSCC (*p* > 0.05).

Notably, 3 patients out of 8 developed OSCC from OLP lesions under fixed prosthesis on implants.

## 4 Discussion

This retrospective study analyzed the general characteristics of a cohort of two hundred and forty‐six patients with Oral Lichen Planus, their clinical progression, and the potential role of various factors in the development of oral squamous cell carcinoma, followed at the Oral Medicine Department of Policlinico Universitario Agostino Gemelli.

The overall malignant transformation rate of OLP in our study was 3.25%, with a mean follow‐up of 55.48 months (SD: 37.0, range: 6–210 months). This rate is higher than what has been reported in other studies [15], including a systematic review published by our group [7], which was 1.37%, but remains lower than the 5.98% of a recent study focused on the erosive type of OLP [14].

The higher malignant transformation rate observed in our cohort compared with other studies [36, 37, 38] (36‐38) may be partially explained by the nature of our institution, which serves as a tertiary referral center with a strong oncological focus. Consequently, many patients are referred from other hospitals or private practices for the evaluation and management of clinically advanced, long‐standing, or histologically suspicious lesions. This referral bias likely contributes to an overrepresentation of high‐risk OLP cases in our population, leading to an apparently higher transformation rate than that reported in general or community‐based cohorts.

In this transformation rate and throughout our analysis, we did not include five patients who presented to our clinic with an oral squamous cell carcinoma (OSCC) diagnosed at the first visit by incisional biopsy, in whom concomitant lesions of oral lichen planus were also observed. This exclusion was applied because it is not possible to ascertain with sufficient certainty whether the OSCC was a direct consequence of oral lichen planus rather than an independent malignancy arising de novo. Accordingly, these cases were considered separately to avoid overestimating the transformation rate attributable to OLP.

After the analysis of our data, results confirmed a female predominance in malignant transformation, with 7 out of 8 patients being women, consistent with the female‐to‐male ratio observed in our full cohort. Our study results align with several studies in the literature [39, 40, 41, 42], even if contrary results were presented by Bandyopadhyay et al. [43], who found a male predilection in OLP (54.55% males vs. 45.45% females) and in the Munde et al. study [44], where the M:F ratio was 1.6:1. Our results point to the multifocal carcinogenic tendency in the subset of transformed OLP patients. The development of multiple metachronous primary OSCCs in 3/8 cases in our series is consistent with the extension of the field cancerization concept to OLP, where chronically inflamed mucosa may harbor widespread molecular alterations that predispose to more than one independent malignant event over time [45, 46]. In parallel, the observation that 3/8 carcinomas arose from OLP lesions located under fixed implant‐supported prostheses raises a clinically relevant issue: peri‐implant tissues can represent a blind spot, where limited visual access, overlapping inflammatory changes (e.g., peri‐implant mucositis/peri‐implantitis), and potential chronic mechanical irritation may delay recognition of early malignant change [47]. Taken together, these findings support a practical message: in OLP patients, especially those with a history of dysplasia or prior OSCC, clinicians should maintain a low threshold for biopsy of any persistent peri‐implant erythema/ulceration or atypical granulation tissue, and ensure meticulous, regular mucosal inspection even in areas partially covered by fixed prostheses [45].

This gender disparity is thought to be influenced by hormonal factors, as the onset or exacerbation of OLP often coincides with menopause [48]. This finding also aligns with existing literature indicating a higher prevalence of OLP and its malignant transformation in females [49, 50, 51].

The mean age of our cohort was 63.2 years, a figure consistent with those previously reported in the literature. Recent papers have estimated a similar mean age for OLP: two population‐based studies [52, 53] and one clinic‐based study focused on geriatric patients aged 60 to 65 years and above [39]. Notably, the average age in the transformed group was higher, at 74.13 years, reinforcing age as a potential risk factor for OSCC development.

Women over 50 years old represent the demographic most affected by OLP, with studies indicating that this age group accounts for the majority of cases [40]. Also, in our cohort the group of patients aged between 70 and 79 was the most represented. We also report the follow‐ups of 15 patients affected by OLP who had less than 35 years. Interestingly, 5 of these patients were under 18 years old. In this young population the most prevalent type of OLP was the reticular and the plaque‐like, and the most hit site was the dorsum of the tongue, as confirmed also in the study on the largest pediatric cohort affected by OLP by Cascone et al. [41].

In our department, patients underwent periodic follow‐up visits two to four times per year, depending on their symptoms and overall clinical status, as recommended by the literature guidelines [42]. An incisional biopsy was performed whenever there were changes in the clinical appearance of the lesions, a general worsening of the disease, or severe symptoms reported by the patient. Thus, the average number of biopsies in the t‐OLP group was 4.63 (*p* < 0.001), with some patients undergoing up to nine procedures. In contrast, non‐transformed patients typically had fewer than two biopsies. This significant difference highlights how clinicians are often able to identify high‐risk lesions through careful clinical observation, prompting repeated histological assessments. The elevated biopsy frequency suggests that lesions with malignant potential frequently exhibit persistent or atypical clinical features, reinforcing the crucial role of vigilant clinical follow‐up and early intervention in managing OLP [50]. Moreover, this finding is justified by the behavior of OLP and OSCC developed from OLP.

In relation to this topic, four of the eight patients who developed carcinoma had previously been diagnosed with mild or moderate dysplasia in oral biopsies of the same transformed sites. Notably, three patients previously diagnosed with dysplasia developed two or more OSCCs. These findings reinforce the concept of OLP as a cancerization field, where the development of one carcinoma increases the likelihood of subsequent malignancies, which is estimated at 11% [16, 54].

Our findings about dysplasia have been previously reported and analyzed in the literature, suggesting a higher tendency towards multiple OSCCs in the OLP/OED (oral epithelial dysplasia) group [55]. This finding, as studied by Lodi et al. [34], seems to confirm that the diagnosis of dysplasia in OLP represents an early stage of oncological transformation of the disease. The overall rate of mild or moderate dysplasia was 17.07%. The presence of oral epithelial dysplasia (OED) in OLP remains controversial. Some authors have proposed excluding cases with dysplasia from the diagnosis of OLP and from estimates of malignant transformation risk [10, 19]. However, a recent critical review by González‐Moles et al. [56], highlighted that this approach likely underestimates the malignancy potential of the disease, since dysplasia is one of the main drivers of progression to OSCC. This is evident in the meta‐analysis by Idrees et al. [57], which showed that excluding dysplasia from OLP diagnostic criteria reduced the estimated transformation rate from 1.2% to 0.44% across more than 12 000 cases.

In line with the conclusions of González‐Moles and colleagues [56], we believe that the development of epithelial dysplasia in OLP reflects a stage in the progressive malignant transformation of this disease. In cases of oral epithelial dysplasia, management and follow‐up were conducted in accordance with the recommendations outlined in the systematic review by Mehanna et al. [58]. Specifically, surgical excision was performed whenever clinically appropriate to reduce the risk of malignant transformation, and all patients were enrolled in a structured surveillance program with periodic follow‐up visits. This approach reflects the evidence that, although excision lowers the probability of progression to carcinoma, long‐term monitoring remains essential due to the persistent risk of recurrence or malignant change over time. Notably, patients who developed epithelial dysplasia or OSCC underwent more biopsies than the patients who did not progress into malignant diseases. In our experience, the decision to repeat a biopsy typically reflects the clinician's ability to recognize lesions that are clinically evolving or potentially progressing toward malignancy: persistent, atypical, or phenotypically changing lesions prompt closer scrutiny and repeated histological sampling. A higher biopsy count should therefore be regarded as a marker of clinical vigilance and lesion suspicion rather than as an intrinsic biological risk factor.

Regarding clinical presentation, red‐type OLP (atrophic and erosive variants) was significantly associated with dysplasia compared with white‐type lesions (OR = 2.238, 95% CI: 1.100–4.554, *p* = 0.026), supporting the concept that erythematous and ulcerative patterns represent more active and high‐risk forms of the disease [59].

The most frequent sites of malignant transformation in OLP were the buccal mucosa and gingiva, followed by the tongue. These findings are consistent with those reported by Tsushima et al. [15] in a recent retrospective study conducted on a population affected by OLP in Japan, which identified similar localization patterns.

As other diseases, OLP has been associated with several autoimmune disorders. In a recent systematic review and meta‐analysis by De Porras‐Carrique et al. [21] a particular association has been evidenced between OLP, diabetes mellitus (PP = 9.41%, 95% CI = 8.16–10.74; OR = 1.64, 95% CI = 1.34–2.00, *p* < 0.001) and thyroid disease (PP = 7.96%, 95% CI = 6.32–9.75; OR = 1.99, 95% CI = 1.60–2.49, *p* < 0.001). In our cohort, a total of 53 patients (21.5%) reported having conditions unrelated to OLP or no systemic diseases, while 62 patients (25.20%) reported the presence of one autoimmune disease. Additionally, 81 patients (32.93%) were affected by two or more autoimmune disorders, mostly diabetes mellitus, thyroid diseases, psoriasis [23] and Sjogren syndrome [60]. These results show a high prevalence of autoimmune diseases in oral lichen planus, compared to the general population: a recent epidemiologic study revealed an incidence of 4.6% of people suffering from one autoimmune disease [61]. However, the presence of comorbidities did not show statistical significance with an increased risk of malignant transformation (*p* = 0.767).

Immunosuppressive therapies, which are meant to be the gold standard in OLP treatment [33], have been suggested as potential triggers for malignant transformation in OLP. However, in our study, the use of topical and/or systemic corticosteroids did not appear to influence the risk of malignant transformation (*p* = 0.160). Additionally, there were no recorded cases of prolonged continuous use of topical steroids over extended periods. Therefore, based on our findings and in agreement with those published by Tsushima et al. [15], steroid therapy does not seem to impact the risk of malignant transformation in OLP patients.

It's well known that patients with OLP show a higher prevalence of *Candida* spp. infection and are more susceptible to developing candidiasis, particularly when undergoing topical or systemic immunosuppressive therapy. However, there is a lack of studies specifically evaluating the real role of Candida in OSCC development from OLP [7]. Candida is known to induce chronic inflammation and can produce carcinogenic N‐nitrosobenzylmethylamine [62], as well as mutagenic levels of acetaldehyde [63]. However, Candida spp. colonization was found in 37.5% of t‐OLP patients and in 22.4% of the overall OLP cohort. Despite its higher prevalence among those who developed OSCC, Candida colonization was not statistically linked to malignant transformation (*p* = 1.000).

Despite this, patients with an incomplete clinical response showed a higher rate of baseline Candida positivity (OR: 2.34, 95% CI 1.18–4.63; *p* = 0.014). This association is not easy to interpret and may simply indicate that Candida flags a subgroup with greater barrier disruption and local immune dysregulation, in whom inflammation is harder to control and opportunistic colonization is easier to establish. Although this observation would be compatible with a role for Candida screening in patients with symptomatic or ulcerative OLP, or those undergoing topical corticosteroids [64], our data do not allow firm conclusions on this point.

A clinical feature worth highlighting is the tendency of OLP lesions, both red (i.e., erosive‐atrophic and bullous) and white (i.e., reticular and plaque‐type), to exhibit hyper‐keratinization after treatment with topical steroids. The observed tendency towards keratinization in Oral Lichen Planus (OLP) lesions following topical corticosteroid therapy may result from a combination of biological and pharmacological mechanisms. Firstly, corticosteroids reduce inflammation by inhibiting pro‐inflammatory cytokines such as IL‐1, IL‐6, and TNF‐α, consequently modulating T‐cell activity [65]. This reduction in inflammation might lead to decreased T‐cell‐mediated cytotoxicity against keratinocytes, allowing an unregulated proliferative response that favors keratinization. In addition to this, as part of an adaptive protective response, the oral mucosa may develop a thicker keratin layer to shield itself from persistent mechanical or chemical irritants, especially in previously inflamed tissues.

Finally, it is worth noting that in our cohort most OLP‐associated OSCCs were detected at an early stage, in line with published comparative series showing that carcinomas arising on OLP lesions are frequently diagnosed as T1–T2 and N0 disease [16], likely reflecting closer surveillance and earlier clinical interception than in unselected oral cavity SCC populations. At the same time, our data also suggest that a small subset can behave aggressively, presenting with locally advanced and/or high‐risk nodal disease despite follow‐up. This “dual pattern” has been noted by authors discussing OLP‐related carcinogenesis within a field cancerization framework, where multifocal mucosal susceptibility can coexist with occasional cases showing loco‐regional spread even under surveillance [41]. Importantly, when regional metastasis occurs, the presence of extranodal extension (ENE) can rapidly upstage nodal status and overall stage in oral cavity SCC, underscoring how biological aggressiveness, rather than delayed access to care alone, may drive advanced presentation in a minority of OLP‐associated tumors [66].

### 4.1 Strengths and Limitations

One of the key strengths of this study lies in its large sample size and long follow‐up period, which is over 17 years for some patients, which provides robust data on the malignant transformation rate of Oral Lichen Planus. Additionally, the study's clinical and histological diagnostic criteria ensured a high level of accuracy in identifying both OLP and malignant transformation events. The detailed analysis of risk factors, including both demographic and clinical data, provides a solid analysis of the main features of OSCC associated with oral lichen planus. Importantly, patient management was delivered by the same clinical team using standardized and reproducible protocols across the study period, which likely reduced inter‐operator variability and enhanced the internal consistency of follow‐up, treatment decisions, and outcome ascertainment.

However, several limitations must be acknowledged. First, the retrospective, single‐centre design carries an inherent risk of information and selection bias. As a tertiary referral centre, our unit is likely enriched for complex, symptomatic, long‐standing, or clinically suspicious OLP cases (referral bias); the reported malignant transformation rate should therefore be interpreted with caution and not extrapolated to the general OLP population. Second, the frequency and duration of follow‐up were not uniform but tailored to individual clinical status, introducing heterogeneity in person‐time at risk. Finally, with only eight OSCC events, a multivariable model for malignant transformation could not be performed, as the number of events per variable fell below the threshold required for stable estimation. Factors associated with transformation were therefore explored only at the univariate level, and the corresponding findings should be regarded as exploratory and hypothesis‐generating.

Prospective, multicentre studies with larger numbers of transformation events, standardized follow‐up, and centralized histopathological review are needed to validate these findings.

## 5 Conclusions

Built on our long‐standing clinical experience, this single‐centre retrospective cohort reflects a standardized and reproducible care pathway applied to all patients with oral lichen planus. In our routine practice, diagnosis is established through consistent clinical and histopathological criteria, a Candida swab is systematically performed at the first visit and when indicated, and biopsy is undertaken at baseline and subsequently repeated approximately every two years, unless earlier re‐biopsy is prompted by suspicious tissue changes. Patients are re‐evaluated at regular 4‐month intervals, providing a structured framework for surveillance.

Within this setting, OLP demonstrated a 3.25% malignant transformation rate over a mean follow‐up of approximately 55 months, while epithelial dysplasia was identified in 17.1% of patients, predominantly of mild–moderate grade. These observations corroborate OLP as an important oral potentially malignant disorder and position dysplasia as a frequent waypoint in its natural history.

Risk factors for dysplasia were consistent, with older age and red‐type clinical variants (atrophic/erosive) showing significant associations. These data support intensified surveillance for lesions that persist, change phenotype, or present with erythema/ulceration.

Focusing on oncological development, malignant transformation was most consistently preceded by a prior dysplastic diagnosis at the same site, underscoring dysplasia as the critical step toward carcinoma within the OLP continuum. Collectively, our findings support a risk‐adapted care pathway: (i) routine follow‐up for all OLP; (ii) short‐interval reviews (e.g., every 3–6 months) with a low threshold for re‐biopsy in red‐type, symptomatic, non‐responder, or clinically evolving lesions; and (iii) long‐term monitoring of any site with prior dysplasia. The retrospective design and the limited number of transformation events constrain causal inference; nonetheless, the internal consistency across analyses strengthens the central message: the trajectory from OLP to OSCC largely proceeds through dysplasia, and vigilant, repeated assessment within a standardized, reproducible clinical protocol remains the most reliable strategy for early interception.

## Funding

The authors have nothing to report.

## Conflicts of Interest

The authors declare no conflicts of interest.

## Supporting information


**Data S1:** Results of the statistical analysis stratified for dysplasia. * Total number of patients receiving OLP treatment.

**Data S2:** (a) Demographic and clinical characteristics of patients who developed OSCC. (b)Disease‐related features and follow‐up.

## Data Availability

The data that support the findings of this study are available from the corresponding author upon reasonable request. The data are not publicly available because they contain information that could compromise the privacy of the study participants, in accordance with institutional and ethical restrictions.
