# Supplementary material for: Long‐Term Care of OLP Patients: A Retrospective Study From a Single Hospital Outpatient Clinic
Source: J Oral Pathol Med. 2026 Sep 4;55(10):1121–36. doi: 10.1111/jop.70179 (PMC13728523; doi:10.1111/jop.70179)
Supplement: Supplementary file 1 [file JOP-55-1121-s001.docx]

**Supplementary Materials 2a.** Demographic and clinical characteristics of patients who developed OSCC

| Patient | Sex | Age (years) | Systemic diseases | Nr. of biopsies | Smoking |
| --- | --- | --- | --- | --- | --- |
| 1 | F | 79 | Hypertension, diabetes, hypercholesterolemia | 5 | Heavy smoker |
| 2 | F | 83 | Hypertension, diabetes, asthma | 2 | No |
| 3 | F | 62 | Osteopenia, hypercholesterolemia | 1 | No |
| 4 | F | 88 | Hypertension, diabetes | 2 | No |
| 5 | F | 56 | None | 1 | No |
| 6 | F | 84 | Hypertension | 3 | No |
| 7 | F | 63 | Diabetes | 2 | No |
| 8 | M | 78 | Ocular thrombosis | 3 | Heavy smoker |

**Supplementary Materials 2b.** Disease-related features and follow-up.

| Patient | Lichen in other districts | Responder to OLP therapy | Site of transformation | | Follow-up (months) | Stage at diagnosis *AJCC VIII Edition* |
| --- | --- | --- | --- | --- | --- | --- |
| 1 | No | Yes | | Ventral tongue and floor of the mouth | 74 | I |
| 2 | No | Yes | | Gingiva | 41 | II |
| 3 | Cutaneous | No | | Gingiva | 33 | IVB |
| 4 | No | Yes | | Buccal mucosa | 63 | IVA |
| 5 | No | Yes | | Gingiva | 36 | I |
| 6 | No | No | | Gingiva, floor of the mouth, buccal mucosa | 26 | I |
| 7 | No | Yes | | Gingiva, floor of the mouth, buccal mucosa | 38 | IVA |
| 8 | No | Yes | | Dorsum of the tongue | 23 | I |
