# Supplementary material for: Long‐Term Care of OLP Patients: A Retrospective Study From a Single Hospital Outpatient Clinic
Source: J Oral Pathol Med. 2026 Sep 4;55(10):1121–36. doi: 10.1111/jop.70179 (PMC13728523; doi:10.1111/jop.70179)
Supplement: Supplementary file 2 [file JOP-55-1121-s002.docx]

| **Variable** |  | **No dysplasia** | **Dysplasia** | **Total** | **Statistical significance** |
| --- | --- | --- | --- | --- | --- |
| Sex | Men | 63 (30.9%) | 10 (23.8%) | 73 (29.7%) |  |
|  | Women | 141 (69.1%) | 32 (76.2%) | 173 (70.3%) | χ2 Test —p=0.459 |
|  | Total | 204 (100%) | 42 (100%) | 246 (100%) |  |
|  |  |  |  |  |  |
| Age (years) | Mean (Range, SD) | 61.37 (13-90; SD: 14.94) | 72.38 (56-88; SD: 11.48) | 63.2 (13-95; SD: 14.98) | **Mann Whitney – p= 0.000** |
|  |  |  |  |  |  |
| Age at diagnosis (decades) | 0-49 | 39 (19.1%) | 1 (2.4%) | 40 (16.3%) | **χ2 Test —p=0.001** |
|  | 50-59 | 37 (18.1%) | 6 (14.3%) | 43 (17.5%) |  |
|  | 60-69 | 56 (27.5%) | 7 (16.7%) | 63 (25.6%) |  |
|  | 70-79 | 53 (26%) | 16 (38.1%) | 69 (28.1%) |  |
|  | Over 80 | 19 (9.3%) | 12 (28.6%) | 31 (12.6%) |  |
|  | Total | 204 (100%) | 42 (100%) | 246 (100%) |  |
|  |  |  |  |  |  |
|  |  |  |  |  |  |
| Follow-up (months) | Mean (Range, SD) | 52.13 (6-210; SD: 35.36) | 71.67 (9-181; SD: 40.83) | 55.48 (6-210; SD: 37.00) | **Mann Whitney – p= 0.002** |
|  |  |  |  |  |  |
| OLP Type | Reticular | 69 (33.8%) | 3 (7.1%) | 72 (29.3%) | **χ2 Test —p=0.001** |
|  | Plaque | 76 (37.3%) | 17 (40.5%) | 93 (37.8%) |  |
|  | Erosive | 37 (18.1%) | 16 (38.1%) | 53 (21.6%) |  |
|  | Atrophic | 20 (9.8%) | 6 (14.3%) | 26 (10.6%) |  |
|  | Bullous | 2 (1%) | 0 (-) | 2 (0.8%) |  |
|  | Total | 204 (100%) | 42 (100%) | 246 (100%) |  |
|  |  |  |  |  |  |
| OLP localization | Gingiva | 121 (49.2%) | 24 (57.1%) | 145 (58.9%) | χ2 Test —p=0.864 |
|  | Buccal mucosa | 131 (64.2%) | 24 (57.1%) | 155 (62.9%) | χ2 Test —p=0.400 |
|  | Tongue | 112 (45.5%) | 26 (61.9%) | 138 (56.1%) | χ2 Test —p=0.495 |
|  | Palate | 72 (35.3%) | 12 (28.6%) | 84 (34.2%) | χ2 Test —p=0.159 |
|  | Mouth floor | 60 (29.4%) | 9 (21.4%) | 69 (28.1%) | χ2 Test —p=0.097 |
|  | Lips | 33 (16.2%) | 3 (7.1%) | 36 (14.6%) | χ2 Test —p=0.105 |
|  |  |  |  |  |  |
| Other districts | Cutaneous | 40 (19.6%) | 3 (7.2%) | 43 (17.5%) | χ2 Test —p=0.225 |
|  | Genital | 10 (4.9%) | 2 (4.8%) | 12 (4.9%) |  |
|  | Both | 9 (4.4%) | 2 (4.8%) | 11 (4.5%) |  |
|  |  |  |  |  |  |
| Smoking | Smoker | 68 (33.3%) | 4 (9.5%) | 72 (29.3%) | **χ2 Test —p=0.001** |
|  | Non smokers | 136 (66.7%) | 38 (90.5%) | 174 (70.7%) |  |
|  | Total | 204 (100%) | 42 (100%) | 246 (100%) |  |
|  |  |  |  |  |  |
| Comorbidities | None | 77 (37.8%) | 18 (42.9%) | 95 (38.6%) | χ2 Test —p=0.858 |
|  | One | 52 (25.5%) | 12 (28.6%) | 64 (26%) |  |
|  | Two or more | 75 (36.8%) | 12 (28.6%) | 87 (35.4%) |  |
|  | Total | 204 (100%) | 42 (100%) | 246 (100%) |  |
|  |  |  |  |  |  |
| Candida spp. swab | Negative | 162 (76%) | 29 (54.8%) | 191 (77.6%) | χ2 Test —p=0.008 |
|  | Positive | 42 (24%) | 13 (45.2%) | 55 (22.4%) |  |
|  | Total | 204 (100%) | 42 (100%) | 246 (100%) |  |
|  |  |  |  |  |  |
| Candida therapy | No | 156 (76.5%) | 23 (54.8%) | 179 (72.8%) | χ2 Test —p=0.668 |
|  | Yes | 48 (23.5%) | 19 (45.2%) | 67 (27.2%) |  |
|  |  | 204 (100%) | 42 (100%) | 246 (100%) |  |
|  |  |  |  |  |  |
| Number of biopsies | Less than 2 | 164 (80.4%) | 17 (40.5%) | 181 (73.6%) | χ2 Test —p=0.000 |
|  | More than 2 | 40 (19.6%) | 25 (59.5%) | 65 (26.4%) |  |
|  | Total | 204 (100%) | 42 (100%) | 246 (100%) |  |
|  |  |  |  |  |  |
| Responder status | Yes | 58 (61.1%) | 15 (65.2%) | 73 (61.9%) | χ2 Test —p=0.794 |
|  | No | 37 (38.9%) | 8 (34.8%) | 45 (38.1%) |  |
|  | Total | 95 (100%) | 23 (100%) | 118 (100%)^*^ |  |

**Supplementary Materials 1.** Results of the statistical analysis stratified for dysplasia. * Total number of patients receiving OLP treatment.
